# 2020 family medicine postgraduate examinations at The University of the West Indies: successes and challenges in the time of COVID-19 pandemic

**DOI:** 10.1136/postgradmedj-2021-140242

**Published:** 2021-05-26

**Authors:** Shastri Motilal, Joanne Paul-Charles, Monika Asnani, Raveed Khan, Tana Ricketts-Roomes, Sabriquet Pinder-Butler, Joseph Herbert, Carnille Farquharson, Catherine Conliffe, Aileen Standard-Goldson, Kristen Smith, Euclid Morris, Rohan G Maharaj

**Affiliations:** Paraclinical Sciences, The University of the West Indies, St. Augustine Campus, Faculty of Medical Sciences, St Augustine, Trinidad and Tobago; The University of the West Indies, Cave Hill Campus, Faculty of Medical Sciences, St Michael, Barbados; Caribbean Institute for Health Research—Sickle Cell Unit, The University of the West Indies at Mona Faculty of Medical Sciences, Mona, Saint Andrew, Jamaica; Paraclinical Sciences, The University of the West Indies, St. Augustine Campus, Faculty of Medical Sciences, St Augustine, Trinidad and Tobago; Department of Community Health and Psychiatry, The University of the West Indies at Mona Faculty of Medical Sciences, Mona, Saint Andrew, Jamaica; University of the West Indies School of Clinical Medicine and Research, Nassau, Bahamas; The University of the West Indies, Cave Hill Campus, Faculty of Medical Sciences, Bridgetown, Saint Michael, Barbados; University of the West Indies School of Clinical Medicine and Research, Nassau, Bahamas; University of the West Indies School of Clinical Medicine and Research, Nassau, Bahamas; Department of Community Health and Psychiatry, The University of the West Indies at Mona Faculty of Medical Sciences, Mona, Saint Andrew, Jamaica; Department of Community Health and Psychiatry, The University of the West Indies at Mona Faculty of Medical Sciences, Mona, Saint Andrew, Jamaica; The University of the West Indies, Cave Hill Campus, Faculty of Medical Sciences, Bridgetown, Saint Michael, Barbados; Paraclinical Sciences, The University of the West Indies, St. Augustine Campus, Faculty of Medical Sciences, St Augustine, Trinidad and Tobago

**Keywords:** education & training (see medical education & training)

## Abstract

Little has been published regarding postgraduate assessments during the COVID-19 pandemic. There is an urgent need to graduate well-trained specialists including family physicians who play a key role in patient care. The successes and challenges encountered in mounting qualifying 2020 Family Medicine examinations during the COVID-19 pandemic at the University of the West Indies are described in this paper. Human resource, planning, use of technology and virtual environments are discussed, which enabled successful examinations at this multicampus regional site.

## Introduction

All facets of medical education have been challenged with the declaration of the novel SARS-COV-2 virus (COVID-19) pandemic in March 2020.^1^ Medical schools have had to adapt and innovate.^2^ Despite documented challenges and solutions in qualifying medical students, there is a paucity of literature on final assessments in postgraduate education during the pandemic.^3–6^ There is a need to graduate specialists during a pandemic to keep up with the evolving healthcare needs of the population. Family Medicine is no exception, as family doctors are expected to fulfil their role of providing continuous, comprehensive, first-contact patient-centred care. As front liners, they are vital in the myriad public health response to COVID-19.^7^

In this context, we describe the successes and challenges encountered in mounting qualifying Family Medicine examinations during the COVID-19 pandemic at the authors’ institution. The University of the West Indies (The UWI). The Family Medicine programmes at The UWI have been in existence for 40 years. Uniquely, we are a multicampus university with teaching centres across four Caribbean islands, with common exit examinations for a Diploma and Doctor of Medicine, in Family Medicine.^8^ The inception, growth and strengths of the Family Medicine programmes as a model for the developing world have been previously described.^8^ The annual cross-campus Family Medicine examinations usually occur at the end of the academic year requiring travel of students and examiners between the sites.

## Family medicine examinations at UWI during the COVID-19 pandemic

The examinations are typically held in May–June each year, but in 2020, The UWI administration decided to postpone examinations due to the pandemic. During the new examination period (24 November to 2 December 2020), there were on average 18, 500, 4387 and 795 active COVID-19 cases on The UWI islands of Barbados, Bahamas, Jamaica and Trinidad and Tobago, respectively.^9^ There were restrictions in place on travel between the islands, and public health measures mandated social distancing, mask wearing, universal hand hygiene and appropriate quarantine at all sites. While some Family Medicine bodies made the decision to cancel 2020 examinations with resulting challenges for credentialling, the decision of The UWI was for postgraduate examinations to occur.^10 11^ The challenge was to mount final assessments that preserved the regionality and integrity of the examination process, yet safe for academic and support staff and students.

The written examinations comprised multiple-choice questions (MCQs) and extended matching questions (EMQs) that were proctored in person by invigilators and staff following strict regulations both at the university and national levels. Selected venues allowed for at least 2 m between candidates. Students completed screenings for elevated temperature and symptoms of COVID-19 infection and prevention measures were enforced at examination sites.

The emphasis of the Family Medicine Objective Structured Clinical Examinations (OSCE) has traditionally tested students’ ability to conduct patient-centred consultations across the spectrum of clinical scenarios typical of first-contact, community-based care. Maintaining such standards while adhering to COVID-19 public health regulations meant transforming this examination into a completely virtual exercise that was challenging. This new concept required significant planning on the practicalities of the examination process while maintaining traditional assessment goals and integrity.^12^ The process involved selection of a familiar online platform, combining and restructuring test skills with reduction of OSCE stations while maintaining blueprinting, designing the OSCE circuit, organising the collaboration of human resources such as examiners, information technology (IT) staff, simulated patients, examination candidates and administrative-technical staff, all these while ensuring comparable standards across all campus sites.

## Maintenance of examination rigour

Table 1 highlights the contrast between previous examination format and the pandemic examinations.

**Table 1:** Modifications made to pandemic examinations

Final assessment	Previous examination format	Pandemic examination format
Diploma written examination	In-person proctored short answer questions and extended matching questions	In-person *proctored multiple choice and extended matching questions
DM written examination	In-person proctored essays, extended matching and multiple choice questions	In-person *proctored multiple choice and extended matching questions
Diploma OSCE Examination	Face to face 12 station OSCE (10 with patients and two slideshows)	†Virtual 10-station OSCE (eight with patients and two slideshows)
DM OSCE Examination	Face to face 5-station OSCE (five patients)	†Virtual 5-station OSCE (five patients)
DM oral examination of research project	Face to face oral presentation	†Virtual oral presentation

* Candidates invigilated while doing paper-based examination at a designated centre under pandemic restrictions.

† Video-conferencing using Zoom.

DM, Doctor of Medicine; OSCE, Objective Structured Clinical Examinations.

Written papers were redesigned using MCQs and EMQs in anticipation of mounting online synchronous examinations. The mandate of The UWI, however, shifted to in-person proctored examinations adhering to all national pandemic protocols. All written papers were blueprinted, as before, for validity based on learning outcomes. Questions on COVID-19 were included. As with past examinations, review and feedback on all written examinations by an external examiner were accomplished using secure password-protected online platforms. OSCE examinations were developed such that all participating sites had a unique examination with different clinical cases. Training of simulated patients was done virtually to ensure reliability in performance. Oral presentations and examination of research projects also occurred via videoconferencing in a secure environment with social distancing restrictions in place. Written and OSCE examinations were all standard set to provide minimal competencies as determined by academics from all sites using modified dichotomous Angoff methods.^13 14^ This ensured all successful candidates met an acceptable standard in both knowledge and practice.

## Use of technology

The use of Zoom video conferencing online software for OSCE examination has been well described.^15 16^ Use of the breakout room feature allowed multiple simultaneous rooms—each with examiners, patients and students. Each person had an internet-ready device with a camera and headset with microphone. Managing the breakout rooms with proper timing and synchronisation was key in ensuring the smooth conduct of the virtual OSCE. WhatsApp (Facebook, USA) instant messenger groups were used for ease of communication between examiners and sites for troubleshooting and secure email was used for transmission of marksheets.

Oral examinations were also part of the postgraduate Family Medicine examinations. These were conducted virtually using Zoom. In addition to the functionalities described above, grading was also done electronically using Google forms. The use of such customisable online forms in creating online marksheets has also been used in the OSCE setting.^17^ It avoids manual scoring and missed checkboxes and allows for immediate grade calculation.

## Pre-existing factors that contributed to success

First, distance education technology had been frequently used by Family Medicine academics prior to the pandemic (eg, in examination preparation) and staff were comfortable and experienced in using it. This situation had been fueled previously by the physical distance between islands.

Second, both Jamaica and Barbados currently conduct a portion of their academic work for rural Jamaica and the Eastern Caribbean by distance education modalities. The physical separation and distance of the archipelago meant that the administration of The UWI had been investing significantly in distance education technology for several years.

Furthermore, there was institutional support embodied by The UWI’s triple-A strategic plan 2017–2022: Access to quality tertiary education, alignment with the needs of the region and agility in response to the ever-changing environment.^18^ With strategic objectives including digital transformation, fostering academic collaboration and ensuring access to university training for Caribbean people in remote areas, this plan ensured that the university was well positioned to face the challenge of mounting these examinations using remote methodologies.

## Human resource and planning

The mounting of such an examination was no easy feat and was only accomplished because of immense preparation and collaboration. A core group of about 14 persons from all four sites held regular meetings using Zoom. All academics, most of whom were familiar with online technologies, were forced to rely more on this modality for teaching and meetings from the onset of the pandemic. The UWI’s plan for digital transformation was agile in staff training and IT support to ensure smooth and timely pivoting to online teaching for those who needed it.^19^ This prepared staff for the new normal of virtual environments, and was key to successfully conducting these examinations.

IT support persons were critical in the smooth conduct of these examinations. They were pooled from all campuses to discuss the feasibility of virtual examinations, and a joint needs analysis was conducted across campuses to determine the specific requirements for all virtual examinations. Key responsibilities of IT specialists included the creation of hosting links, configuring devices, troubleshooting and breakout room management. Virtual simulation exercises with IT specialists allowed for troubleshooting. Students were kept abreast of the proposed changes and participated in virtual practice runs to familiarise them with the technology. Virtual training of the simulated patients also minimised physical contact.

## Successes

In addition to the maintenance of assessment validity and reliability, there were also several noteworthy achievements. A total of 53 candidates were examined with 48 successfully completing their examinations, a pass rate (91%) comparable to that of pre-COVID-19 years. All virtual clinical examinations ran on time with few technical glitches and without any breaches in local COVID-19 regulations. Virtual breakout rooms allowed for the shuffling of students without them leaving their assigned individual physical space as obtained in face-to-face examinations. It also meant that students, patients and examiners could communicate unhindered without a mask inside their enclosed spaces. Virtual rooms allowed examiners from one campus to examine candidates from another, off-site examination of candidates as well as examiner meetings.

The absence of all examiner-related travels in 2020 would have translated into reduced costs of air travel and accommodation. It also meant more efficient use of examination time. An independent external examiner would usually visit to observe the entire examination process as a quality control measure to maintain examination integrity. The virtual environment allowed the external examiner to fulfil this role having observed all candidates and all OSCE scenarios at all four sites. Usual examination rules of no personal electronic devices and communication were also enforced at all physical sites by invigilators.

Candidates, simulated patients and, in some cases, examiners were brought to a central location at each site. They were each housed in a separate room/space in accordance with COVID-19 protocols. This allowed invigilator oversight of candidates and IT presence for troubleshooting and on-hand support for all. IT personnel had the opportunity to work collaboratively in the planning process, affirming our concept of operating as The One UWI.^20^

## Challenges and proposed solutions

The main challenge faced in mounting these examinations was the heavy dependence on technology and IT support. The initial investment in purchasing additional devices and equipment may, however, prove to be cost-effective as this mode of examination may be required in the medium term. Power losses and loss of connectivity were also possible threats. Examination venues with power backup and redundant internet hotspots were some of the contingencies in place to cater for these eventualities. Fortunately, major internet disruptions were not common during the examinations, and the minor lapses in audio and video quality that occurred did not detract significantly from the point of view of the participants. Training of backup patients, having extra examiners on standby and having backup physical spaces with ready devices, were some of the contingencies in place to avoid unplanned mishaps.

The inability to reliably test physician skills at physical examinations on real patients was a limitation. Structuring OSCE scenarios where examination findings are elicited by questioning, while not a replacement for a physical examination, may test integration of clinical findings. Observing candidates perform examinations during their training until a defined competency level is attained, and formative in-house clinical skills assessments may also negate the need to test such a skill in an exit examination.

## Next steps and conclusions

Future research involving the students, simulated patients and examiners from all sites may prove useful. The perspective of all parties who participated in the Family Medicine examinations may produce useful insights into how the above examination experience described can be improved. Due to worldwide changes in modes of learning, training programmes should incorporate development of basic IT competencies as an ongoing requirement for their faculty. This will help mitigate future disruptions to face-to-face training and assessments and can take the form of online modules, Continuing Medical Education or onsite instructions. These IT competencies will serve to build capacity not only for assessment and examinations but also for the development of Telehealth capabilities which will surely play a crucial role in the delivery of clinical services and training.

The mandate of UWI to mount clinical postgraduate examinations in the face of a pandemic with so many uncertainties seemed a daunting task initially. In the words of Fuller *et al*, COVID-19 may have led some to experience ‘there is no alternative’ moment in assessment, but through collegiality, communication, flexibility and compassion, we turned this challenge into an opportunity.^21^ A virtual OSCE provided an opportunity to test telemedicine skills, which has been a highlight among family physicians more so, since the pandemic. This paper highlights that through innovative approaches involving appropriate use of technology, postgraduate Family Medicine qualifying examinations can be successfully conducted during a pandemic. In these challenging times, family doctors are needed now more than ever.

**List of learning points**

- Family Medicine certification examinations can be successfully conducted virtually during a pandemic.
- Information technology support and redundancies and use of videoconferencing software are necessary for delivering virtual clinical examinations.
- Careful planning, collaboration, human resource and institutional investment are key to ensure smooth running of examinations across distant multicampus sites.
